# Involvement of the miR-137-3p/CAPN-2 Interaction in Ischemia-Reperfusion-Induced Neuronal Apoptosis through Modulation of p35 Cleavage and Subsequent Caspase-8 Overactivation

**DOI:** 10.1155/2020/2616871

**Published:** 2020-12-10

**Authors:** He Wang, Qian Yu, Zai-Li Zhang, Hong Ma, Xiao-Qian Li

**Affiliations:** ^1^Department of Anesthesiology, First Affiliated Hospital, China Medical University, Shenyang, 110001 Liaoning, China; ^2^Department of Thoracic Surgery, Fourth Affiliated Hospital, China Medical University, Shenyang, 110032 Liaoning, China

## Abstract

**Background:**

Neuron survival after ischemia-reperfusion (IR) injury is the primary determinant of motor function prognosis. MicroRNA- (miR-) based gene therapy has gained attention recently. Our previous work explored the mechanisms by which miR-137-3p modulates neuronal apoptosis in both *in vivo* and *in vitro* IR models.

**Methods:**

IR-induced motor dysfunction and spinal calpain (CAPN) subtype expression and subcellular localization were detected within 12 h post IR. Dysregulated miRs, including miR-137-3p, were identified by miR microarray analysis and confirmed by PCR. A luciferase assay confirmed CAPN-2 as a corresponding target of miR-137-3p, and their modulation of motor function was evaluated by intrathecal injection with synthetic miRs. CAPN-2 activity was measured by the intracellular Ca^2+^ concentration and mean fluorescence intensity *in vitro*. Neuronal apoptosis was detected by flow cytometry and TUNEL assay. The activities of p35, p25, Cdk5, and caspase-8 were evaluated by ELISA and Western blot after transfection with specific inhibitors and miRs.

**Results:**

The IR-induced motor dysfunction time course was closely associated with upregulated expression of the CAPN-2 protein, which was mainly localized in neurons. The miR-137-3p/CAPN-2 interaction was confirmed by luciferase assay. The miR-137-3p mimic significantly improved IR-induced motor dysfunction and decreased CAPN-2 expression, even in combination with recombinant rat calpain-2 (rr-CALP2) injection, whereas the miR-137-3p inhibitor reversed these effects. Similar changes in the intracellular Ca^2+^ concentration, CAPN-2 expression, and CAPN-2 activity were observed when cells were exposed to oxygen-glucose deprivation and reperfusion (OGD/R) and transfected with synthetic miRs *in vitro*. Moreover, double fluorescence revealed identical neuronal localization of CAPN-2, p35, p25, and caspase-8. The decrease in CAPN-2 expression and activity was accompanied by the opposite changes in p35 activity and protein expression in cells transfected with the miR-137-3p mimic, roscovitine (a Cdk5 inhibitor), or Z-IETD-FMK (a caspase-8 inhibitor). Correspondingly, the abovementioned treatments resulted in a higher neuron survival rate than that of untreated neurons, as indicated by decreases in the apoptotic cell percentage and p25, Cdk5, caspase-8, and caspase-3 protein expression.

**Conclusions:**

The miR-137-3p/CAPN-2 interaction modulates neuronal apoptosis during IR injury, possibly by inhibiting CAPN-2, which leads to p35 cleavage and inhibition of subsequent p25/Cdk5 and caspase-8 overactivation.

## 1. Introduction

Spinal cord ischemia-reperfusion (IR) injury occurs during operations that require a transient block of blood flow to the spinal cord [1, 2]. Usually, reperfusion cannot prevent ischemia-induced neurological impairment (known as primary insults), but it will further aggravate neurological function (known as secondary insults) during the initial period [3]. Apart from the high incidence of sensory deficits, IR injury is reported as a major cause of permanent motor dysfunction due to extensive neuronal death after recovery of blood flow [4–6]. Due to the limited proliferative capacity of adult neurons, exploration of the underlying mechanisms is of particular importance to prevent neuronal death [7]. Various types of neuronal death have been reported, including apoptosis, necroptosis, and ferroptosis [5, 7, 8]. We previously identified which types of cell death are involved in a specific type of spinal cord IR injury and found that blocking apoptosis effectively preserved hind-limb motor function in rodent models [5, 9]. Some recent studies have shown that phenomena disturbing ionic homeostasis, such as excessive intracellular calcium ion concentrations ([Ca^2+^]) in neurons induced by ischemic or mechanical injury, could eventually trigger neuronal apoptosis by influencing vital biological functions and metabolism [10–12]. Thus, preserving intracellular calcium homeostasis may represent a promising strategy for attenuating neuronal apoptosis after IR insult.

Increased intracellular Ca^2+^ levels can activate a variety of proteases [13]. Belonging to a family of calcium-dependent neutral proteases, calcium-activated neutral proteinases (CAPNs, also called calpains) are the most well-known effectors that react to intracellular Ca^2+^ dysregulation through calcium-binding subunits [14, 15]. Eleven types of calpain isoforms have been identified in humans thus far, of which calpain-1 (*μ*-calpain (CAPN-1)) and calpain-2 (m-calpain (CAPN-2)) are the most widely ubiquitous isoforms in the central nervous system (CNS) [13]. Exhibiting the same subcellular localization (cytoplasm) and sharing a common small subunit (known as CAPN-4), CAPN-1 and CAPN-2 appear to have similar biochemical properties [13, 16], although they require micromolar and millimolar calcium levels for activation, respectively [13–16]. However, in contrast to traditional views, some studies have recently suggested that CAPN-1 activation plays a prosurvival role while CAPN-2 plays neurodegenerative roles based on their opposite functions in regulating neuronal plasticity following CNS injury [17–19]. Commonly, the proteolytic cleavage products of CAPN-mediated truncation substrates are bioactive [13, 20, 21]. For example, the membrane-bound protein p35 has been demonstrated to be a major substrate exclusively regulated by CAPNs and can further amplify neurotoxic insults or oxidative stress by activating cyclin-dependent kinase-5 (Cdk5) in the pathogenesis of neurodegenerative disease [13, 19, 20]. *In vivo* (rodent) and *in vitro* experiments revealed that overexpressed CAPN-2 precisely cleaved the normally membrane-bound p35 into the more stable p25 form, which finally led to inappropriate increases in p25/Cdk5 activation and protein levels of caspase-3, a final executioner of neuronal apoptosis [19, 22, 23]. Previous structural experiments further identified the N terminus of p35 as the major element necessary to preserve the intact covalent bond within the p35-caspase-8 crystal structure [25]. Consistently, the p35 protein from baculovirus effectively blocked the apoptosis cascade by forming a p35-caspase-8 complex via a thioester bond [24, 25]. Thus, in addition to modulating caspase-3, the final executioner of apoptosis, p35 might mediate the activation of all caspases by regulating caspase-8 proteolysis [26, 27]. Based on this evidence, it is reasonable to infer that increased CAPN-2-mediated p35 cleavage may lead to conformational changes in p35 and subsequently initiate caspase-8 and downstream caspase activation during IR injury.

MicroRNAs (miRs) are a group of small, endogenous, noncoding RNAs [28] that are widely expressed in the CNS and are able to negatively regulate target genes by either degradation or posttranscriptional repression [5, 6, 28]. In our previous studies, we identified hundreds of aberrant miRs in injured spinal cords by microarray analysis [5, 6, 29]. Intrathecal pretreatment with a synthetic miR mimic significantly improved neurological deficits by recovering the altered miR expression [5, 6, 29]. These findings suggest promising miR-based gene therapy targeting CAPN-2. In this context, we first searched bioinformatical databases and identified potential miRs that may bind CAPN-2 among all dysregulated miRs detected by microarray analysis. Our present study results suggested that miR-137-3p and miR-124-3p have target interactions with CAPN-2, which is supported by another study that explored the roles of miR-137-3p in rescuing motor neuron degeneration after brachial plexus root avulsion injury [30]. Then, we studied the functions and mechanisms by which the miR-137-3p/CAPN-2 interaction regulates neuronal apoptosis by pretreatment of *in vivo* and *in vitro* models with synthetic miRs, a selective CAPN-2 inhibitor, recombinant rat calpain-2 (rr-CALP2), or a specific caspase-8 inhibitor.

## 2. Materials and Methods

### 2.1. Experimental Animals

Sprague-Dawley rats weighing 200 to 250 g were obtained from the Animal Center of China Medical University (Shenyang, China). All rats were preacclimatized 7 days before surgery and housed in standard cages under a 12 h light/dark cycle with a temperature of 23-24°C and humidity of 40-50%. The experiments were performed in accordance with the Guide for the Care and Use of Laboratory Animals (United States National Institutes of Health publication number 85-23, National Academy Press, Washington DC, revised 1996).

### 2.2. Rat IR Model Establishment and Experimental Groups

The rat IR model was established by occluding the aortic arch for 14 min [4, 29]. Briefly, after being anesthetized, the rats were catheterized at the left carotid artery and the tail artery to measure proximal and distal blood pressure (BP), respectively. Following exposure of the aortic arch, the clamp was placed between the left common carotid artery and the left subclavian artery for 14 min to induce ischemia, which was confirmed as a 90% decrease in distal BP. Then, the clamp was removed to induce reperfusion for 12 h. The sham-operated rats were subjected to the same procedures except for the induction of ischemia.

### 2.3. miR Microarray Analysis

As we previously reported, rat miRNA microarray analysis was performed with the miRCURY™ LNA Array (version 11.0; Exiqon, Vedbaek, Denmark) [29, 31]. The L_4–6_ segments of the spinal cord were collected at 4 h after reperfusion. According to the manufacturer's instructions, 2.5 *μ*g of total RNA was first labeled with the miRCURY™ Hy3™/Hy5™ Power labeling kit and then hybridized on a miRCURY™ LNA Array (version 18.0; Exiqon, Vedbaek, Denmark).

After removing nonspecifically bound proteins, the microarray slides were scanned by an Axon GenePix 4000B Microarray Scanner (Axon Instruments, CA, USA) for fluorescence detection, and the fluorescence intensities of the scanned images were loaded into the GenePix Pro 6.0 program (Axon Instruments) for feature extraction. The averages of the replicated miRs with intensities of 50 or more were used to calculate a normalization factor. After normalization by the median normalization method, the significantly different miRs were identified by volcano plot filtering. Finally, hierarchical clustering was performed to determine the differences in miR expression by MEV software (version 4.6, TIGR).

### 2.4. Intrathecal Injection and Drug Delivery

All treatments *in vivo*, including synthetic miRs (Dharmacon, Chicago, IL, USA) and recombinant rat calpain-2 (rr-CALP2, B71107, 150 U/L, Calbiochem, China), were diluted to 20 *μ*L in total volume and intrathecally injected, as we previously described [5, 6]. Briefly, the needle of a 25 *μ*m microsyringe was inserted into the L_5–6_ spinal cord segment by the sign of a tail flick. Then, 100 *μ*mol/L miR-137-3p mimic, 125 *μ*mol/L miR-137-3p inhibitor, or 100 *μ*mol/L negative control (NC) was coadministered with Lipofectamine 3000 (Invitrogen, USA) at 24 h intervals for five consecutive days before surgery. Likewise, rr-CALP was dissolved to a final concentration of 75 U/L immediately before injection. The overall effects of the number of treatment days and the dosage used in this study were evaluated by PCR and Western blotting in preliminary experiments. Only the rats that displayed normal motor function were included for further study.

### 2.5. Motor Function Assessment

After being fully preacclimatized to the testing environment, the hind-limb motor functions were scored by the Tarlov system by two observers who were double-blinded to the method [5].

### 2.6. Luciferase Reporter Assay

The target interaction between miR-137-3p and CAPN-2 was verified by a luciferase reporter assay [5]. Briefly, 293T cells were seeded in a 96-well plate at 4 × 10^4^ cells/well and then cotransfected with 100 nM miR-137-3p mimic or 100 nM NC and 180 ng of a luciferase reporter vector containing the wild-type (WT) 3′ untranslated region (3′-UTR) (5′-ACATCGTCTCTCAT*AGCAATA*T-3′) or mutant (MT) 3′-UTR (5′-ACATCGTCTCTCAT*CAUGGCA*T-3′) using Lipofectamine 3000. At 48 h after transfection, the relative activity was determined with a Dual-Luciferase Reporter Assay Kit (Promega Corp., WI, USA).

### 2.7. Oxygen-Glucose Deprivation and Reperfusion (OGD/R) Model

As we previously described, the OGD/R model was established in 70–80% confluent VSC4.1 neurons to mimic IR insult *in vivo* [5]. After two washes and replacement of the medium with glucose-free Hank's balanced salt solution (HBSS), the neurons were kept in an anaerobic chamber (95% N_2_ and 5% CO_2_) at 37°C for 6 h. Then, the initial medium and air conditions were reapplied for another 18 h to induce reoxygenation. The control neurons were cultured in normal and atmosphere conditions for 24 h without deprivation of oxygen or glucose.

### 2.8. VSC4.1 Motor Neuron Culture and Treatments

VSC4.1 motor neurons were purchased from Huatuo Biotechnology Co., Ltd. (Shanghai, China). According to the manufacturer's instructions, the cells were grown in 75 cm^2^ flasks containing 6 mL of culture medium (89% Eagle's minimum essential medium (EMEM) supplemented with 10% fetal bovine serum (FBS) and 1% penicillin/streptomycin) at 37°C with 5% CO_2_ in humidified air. The culture medium was replaced twice weekly.

For the *in vitro* experiment, the neurons were pretreated with synthetic miR and specific inhibitors 24 h before OGD/R insult [5]. As we previously described, after seeding at a concentration of 4 × 10^5^ cells per well, the miR-137-3p mimic (50 nmol/L) or NC (50 nmol/L) was cotransfected with 5 *μ*L of Lipofectamine 3000; for the inhibitor experiments, roscovitine (10 *μ*M, Cdk5 inhibitor, Sigma-Aldrich Co., China) or Z-IETD-FMK (50 *μ*M, caspase-8 inhibitor, R&D Systems, United States) was added to the culture medium alone. The concentration of each treatment agent and the *in vitro* effects were determined by PCR in preliminary experiments.

### 2.9. Detection of CAPN-2 Activity

The tensin homolog (PTEN), a selective CAPN-2 substrate, is degraded by CAPN-2 activation and is widely used for the quantitative analysis of neuronal CAPN-2 activity *in vivo* and *in vitro* [19, 32]. As previously described, CAPN-2 enzymatic activity was assessed by the fold change in the mean fluorescence intensity (MFI) of PTEN (Santa Cruz Biotechnology, CA, USA). The increase in CAPN-2 activity was defined as the MFI in the treated group subtracted from that in the control group. Total CAPN-2 activity was defined as the sum of the MFI in the treated and control groups.

### 2.10. Detection of Cytosolic [Ca^2+^]

The intracellular [Ca^2+^] in VSC4.1 neurons was measured with the Ca^2+^-sensitive indicator Fura-2/acetoxymethyl ester (AM) (Molecular Probes, CA, USA) [14]. After each treatment, the neurons were loaded with 5 *μ*M Fura-2-AM for 30 min at 37°C in the dark. After dilution to 1 × 10^6^ cells/mL with the same Ca^2+^ buffer solution, Fura-2-AM was excited at wavelengths of 340 and 380 nm. The relative changes in intracellular [Ca^2+^] were determined by the fluorescence ratio (*R*) at 340/380 with the following formula: [Ca^2+^] = Kd × *β* × (*R* − *R*_min_)/(*R*_max_ − *R*) [14]. The Calcium Calibration Buffer Kit with Magnesium (Molecular Probes, CA, USA) was used to determine that the Kd, a cell-specific constant, for VSC4.1 neurons was 0.264 *μ*M.

### 2.11. TUNEL Assay

To determine apoptosis in VSC4.1 neurons following OGD/R injury, a TUNEL assay was performed with the Apoptosis Detection Kit (Boster, Wuhan, China). The assay was carried out completely according to the manufacturer's protocol. TUNEL was visualized with DAB staining. The apoptotic neurons were those with either tightly clustered brown staining or more diffuse brown TUNEL staining confined within the cell. With the microscope under a 20x objective, at least 1000 neurons from six random fields in each group were chosen to quantify the total of the TUNEL-positive cells.

### 2.12. Detection of Caspase-8 Activity

Caspase-8 activity was detected by a caspase-8 assay kit (Abcam, CA, USA), which is based on the spectrophotometric detection of the p-nitroaniline (*p*NA) moiety after it is cleaved from the labeled substrate Ac-IETD by caspase-8. The samples were measured in triplicate at an absorbance of 405 nm.

### 2.13. Detection of p25/Cdk5 and p35/Cdk5 Activities by ELISA

Commercialized ELISA kits (Runyu Biological Technology Co., Shanghai, China) were used to measure p25/Cdk5 and p35/Cdk5 activities in VSC4.1 neurons. According to the manufacturer's instructions, the activities in supernatants after each treatment were measured at 450 nm. Each sample was analyzed in triplicate, and the average is presented as ng/L.

### 2.14. Detection of Neuronal Apoptosis by Flow Cytometry

Apoptotic neurons were detected by a BD FACSCalibur flow cytometer (BD Biosciences, MA, USA) at excitation and emission wavelengths of 488 nm and 530 nm, respectively [5]. Briefly, 1 × 10^5^ neurons were first stained with 10 *μ*L of Annexin V-fluorescein isothiocyanate (FITC) at 37°C for 15 min and then counterstained with 5 *μ*L of propidium iodide (PI) for 30 min in the dark. The fluorescence was excited at 488 nm and emitted at 530 nm. Each sample was prepared in triplicate.

### 2.15. Quantitative RT-PCR

Total RNA was extracted from L_4–6_ segments of spinal cords or VSC4.1 neurons by the TRIzol/chloroform method or the miRNeasy FFPE kit (Qiagen, Hilden, Germany) [5]. RNA (500 ng) was reverse transcribed into cDNA by using cDNA SuperMix (TaKaRa, China) or a MicroRNA Reverse Transcription Kit (Applied Biosystems, USA). The levels of miR-137-3p and CAPN-2 were quantified with a TaqMan MicroRNA Assay Kit or a Power SYBR Green PCR Master Mix (TaKaRa, China) on an Applied Biosystems 7500 RT-PCR system (Applied Biosystems, CA, USA). *β*-Actin or U6 was used as an internal control, and each sample was measured in triplicate by the 2^−ΔΔCT^ method. The primers used in this study were as follows: miR-137-3p (forward: 5′-ACACTCATTATTGCTTA-3′; reverse: 5′-CTACGCGTATTGAGAGTAC-3′); CAPN-1 (forward: 5′-CTCCGGGGCAGGAGTAGGCA-3′; reverse: 5′-CTCCGGGGCAGGAGTAGGCA-3′); CAPN-2 (forward: 5′-CTCCGGGGCAGGAGTAGGCA-3′; reverse: 5′-AACTGGCTGTGGGGCTCCCA-3′); U6 (forward: 5′-CTCGCTTCGGCAGCACA-3′; reverse: 5′-AACGCTTCACGAATTTGCGT-3′); and *β*-actin (forward: 5′-GGAGATTACTGCCCTGGCTCCTA-3′; reverse: 5′-GACTCATCGTACTCCTGCTTGCTG-3′).

### 2.16. Double Immunofluorescence (IF)

As previously described [4, 5], for *in vivo* samples, the 20 *μ*m thick spinal cord sections were blocked with 10% bovine serum albumin (BSA) for 1 h and then incubated with the primary mouse anti-calpain-2 antibody (Santa Cruz Biotechnology, sc-373967, 1 : 300, Dallas, USA) and the antibodies specific for neurons (rabbit anti-NeuN, Abcam, ab177487, 1 : 500), astrocytes (rabbit anti-glial fibrillary acidic protein (GFAP), Abcam, ab7260, 1 : 500), and microglial cells (rabbit anti-Iba-1, Abcam, ab178847, 1 : 400) overnight at 4°C. Then, the sections were incubated with Alexa 594-conjugated donkey anti-mouse IgG (1 : 500, Life Technologies, CA, USA) and Alexa 488-conjugated donkey anti-rabbit IgG (1 : 500, Life Technologies, CA, USA) for 2 h at room temperature.

For *in vitro* samples, after being fixed with 4% formaldehyde for 20 min at 4°C, the neurons were permeabilized with 0.1% Triton X-100 for 10 min and blocked with 3% donkey serum for 1 h at room temperature. Then, the neurons were incubated with a primary rabbit anti-p35 antibody (Abcam, ab64960, 1 : 300, CA, USA), primary rabbit anti-TPPP/p25 antibody (Abcam, ab92305, 1 : 300, CA, USA), mouse anti-calpain-2 antibody, or mouse anti-caspase-8 p18 antibody (Santa Cruz Biotechnology, sc-393776, 1 : 400, Dallas, USA) overnight at 4°C and then with Alexa-conjugated secondary antibodies (1 : 500, Life Technologies, CA, USA) for 1 h at room temperature in the dark. For cell counting, the nuclei were counterstained with 4,6-diamidino-2-phenylindole (DAPI, Beyotime Biotechnology, China) for 10 min at room temperature. The images were captured with a Leica TCS SP2 fluorescence microscope (Leica Microsystems, IL, USA), and the integrated fluorescence densities were measured with Leica IM50 software (Nussloch, Germany).

### 2.17. Western Blotting

The total proteins from L_4–6_ spinal cords or VSC4.1 neurons were extracted and purified with a protein extraction kit (KangChen, China) [4, 6]. After determination by a BCA protein assay kit (Beyotime Biotechnology, China), equal protein concentrations were loaded onto a 10% SDS-PAGE gel and transferred to PVDF membranes. The membranes were incubated with 5% skim milk for 1 h to avoid nonspecific binding and probed with an anti-calpain-1 antibody (Santa Cruz Biotechnology, sc-271313, 1 : 400, Dallas, USA), anti-calpain-2 antibody (1 : 500), anti-p35 (1 : 400), anti-TPPP/p25 antibody (1 : 500), anti-PTEN antibody (Santa Cruz Biotechnology, sc-7974, 1 : 400, Dallas, USA), anti-Cdk5 antibody (Santa Cruz Biotechnology, sc-6247, 1 : 300, Dallas, USA), anti-caspase-8 p18 antibody, anti-caspase-3 antibody (Abcam, ab184787, 1 : 500, CA, USA), or *β*-actin (Santa Cruz Biotechnology, sc-47778, 1 : 2000, Dallas, USA) overnight at 4°C. After washing, the membranes were incubated with peroxidase-conjugated secondary antibodies (Beyotime Biotechnology, A0192, 1 : 10,00, China) for 2 h at room temperature. The blots were detected by an ECL kit (Beyotime Biotechnology, China) and quantified by Quantity One software (Bio-Rad Laboratories, Italy).

### 2.18. Statistical Analysis

The data are expressed as the mean ± standard deviation (SD) and were analyzed using SPSS 19.0 software (SPSS, Chicago, USA). Statistical comparisons between two groups were assessed by *t*-tests or the Mann–Whitney tests, whereas comparisons among three or more groups were determined by one- or two-way ANOVA followed by the Tukey-Kramer test. A *P* value < 0.05 was considered statistically significant.

## 3. Results

### 3.1. Temporal Changes in Motor Dysfunction and Spinal CAPN Subtype Expression Post IR

**Figure 1:**
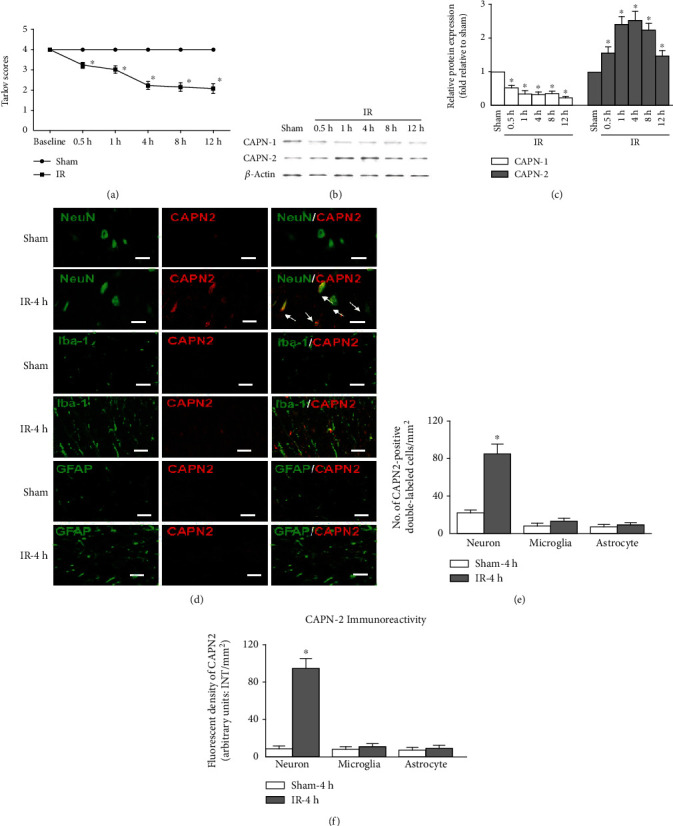
Temporal changes in motor dysfunction and spinal CAPN subtype expression post IR. (a) Temporal changes in hind-limb motor function were evaluated using Tarlov's scores at 0.5, 1, 4, 8, and 12 h after IR. *n* = 6 per group. (b) Representative Western blots of CAPN-1 and CAPN-2 in injured spinal cord samples after IR. (c) Protein quantification of CAPN-1 and CAPN-2 levels after IR. The relative protein levels were normalized to those in the sham group. The data are expressed as the mean ± standard deviation (SD). *n* = 6 per group. ^∗^*P* < 0.05 versus the sham group. (d) Representative double immunofluorescence staining of CAPN-2 with spinal neurons (NeuN), microglia (Iba-1), and astrocytes (glial fibrillary acidic protein (GFAP)) in the anterior horns of spinal cords at 4 h after IR. The yellow labels with white arrows indicate colocalization. Scale bars = 50 *μ*m. *n* = 6 per group. (e, f) Quantification of cells double-labeled with CAPN-2 immunoreactivity and markers of specific cell types. The data are presented as the average of three independent images of laminae II and III in the gray matter and expressed as the mean ± SD. ^∗^*P* < 0.05 versus the sham group.

All rats exhibited normal motor function before undergoing IR surgery. As shown in Figure 1(a), compared with sham-operated rats, the rats in the IR groups displayed obvious hind-limb motor dysfunction, indicated by significant decreases in the average Tarlov score throughout the reperfusion period (*P* < 0.05). Likewise, the protein levels of spinal CAPN-1 and CAPN-2 were measured at 4 h intervals. In contrast to the decrease in CAPN-1 protein expression over time, CAPN-2 protein expression increased unimodally, peaking at 4 h post surgery (Figures 1(b) and 1(c), *P* < 0.05). Notably, there were no significant differences in CAPN-1 protein expression among the IR groups (*P* > 0.05). Thus, the specific cellular localization of CAPN-2 in injured spinal cords was further identified by double immunofluorescence at the time point with the highest CAPN-2 expression. Colocalization was indicated by a yellow fluorescent signal, revealing that the majority of CAPN-2 fluorescent signals overlapped with neurons, not astrocytes or microglia, at 4 h post surgery (Figure 1(d)). Similarly, the quantification of the number of CAPN-2- and NeuN-double-positive cells confirmed that the IR insult-induced increase in CAPN-2 expression was primarily localized in neurons (Figures 1(e) and 1(f), *P* < 0.05).

### 3.2. IR Altered Spinal miR-137-3p Expression and Negatively Regulated CAPN-2 Expression *In Vivo*

**Figure 2:**
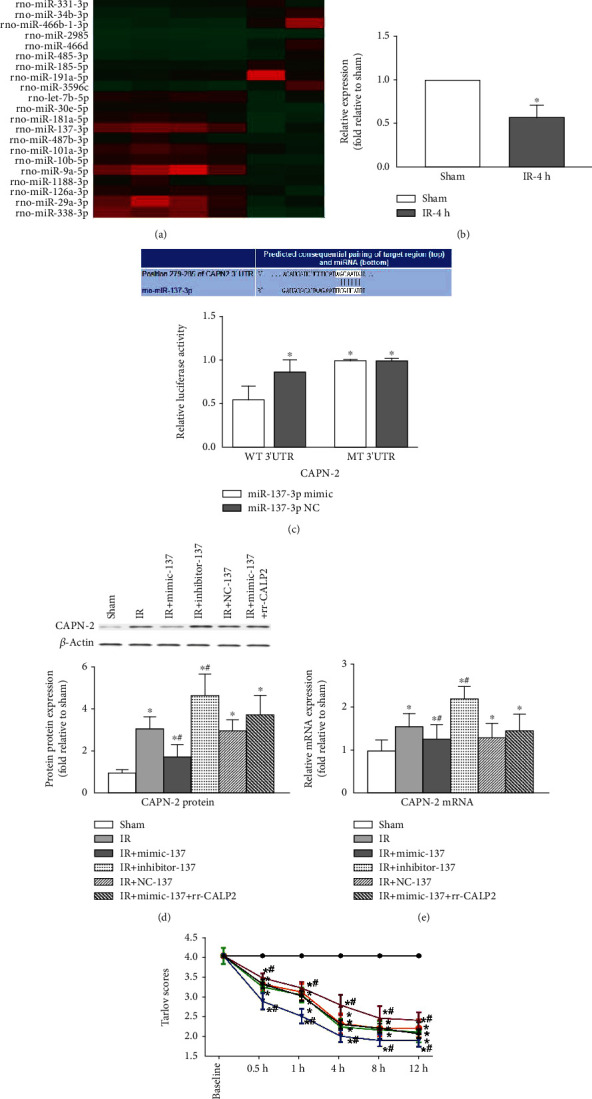
IR induced aberrant spinal miR-137-3p expression and negatively regulated CAPN-2 expression *in vivo*. (a) A heat map representation of miRs differentially expressed in spinal cord samples at 4 h post IR. Three independent replicates were performed. The red signals indicate the upregulated miRs, and the green signals indicate the downregulated miRs. (b) Quantification of miR-137-3p expression post IR. *n* = 4 per group. The data are expressed as the mean ± SD. ^∗^*P* < 0.05 versus the sham group. (c) The putative target binding site of miR-137-3p in the rat 3′-UTR of CAPN-2 was predicted by the TargetScan database and confirmed by a luciferase reporter assay. ^∗^*P* < 0.05 versus the WT 3′-UTR cells transfected with the miR-137-3p mimic. (d) Representative Western blots and protein quantification of CAPN-2 in the spinal cord after different treatments. *β*-Actin was used as a loading control. (e) Quantification of CAPN-2 mRNA expression after different treatments. (f) Hind-limb motor function was assessed by Tarlov's scores after different treatments. ^∗^*P* < 0.05 versus the sham group; ^#^*P* < 0.05 versus the IR group.

Microarray analysis showed that several aberrant miRs were substantially dysregulated in injured spinal cords at 4 h post IR (Figure 2(a)). Among these miRs, miR-137-3p has been indicated to be closely associated with neurodevelopment and CNS diseases and to be highly expressed in the brain [30, 33]. Thus, we hypothesized that miR-137-3p was also widely expressed in spinal cord tissues and confirmed that it showed abnormally decreased expression at 4 h post IR by RT-PCR (Figure 2(b), *P* < 0.05). Moreover, analysis with TargetScan (Release 7.2, http://www.targetscan.org/vert_72/) showed that the miR-137-3p binding site has 7 base pairs that matched the 3′-UTR of the CAPN-2 mRNA. This negative target interaction was further confirmed by a luciferase reporter assay, in which the miR-137-3p mimic significantly decreased the luciferase activity in cells containing the WT 3′-UTR but not the MT 3′-UTR (Figure 2(c), *P* < 0.05), and there were no obvious changes in the luciferase activities of the WT and MT 3′-UTRs of the reporter vector upon cotransfection with the miR-137-3p NC (*P* > 0.05). As we previously reported, the potential *in vivo* interactions were assessed by intrathecal pretreatment with synthetic miRs [5, 6]. Consistently, compared with the IR group, the group intrathecally administered with the miR-137-3p mimic had significantly lower CAPN-2 protein and mRNA expression levels, whereas the group pretreated with miR-137-3p inhibitor injection had significantly higher CAPN-2 expression (Figures 2(d) and 2(e), *P* < 0.05). As expected, the synergistic upregulation in CAPN-2 expression post IR that occurred after injection of rr-CALP2, a recombinant CAPN-2 that specifically upregulates CAPN-2 expression, was partially reversed by miR-137-3p mimic injection (*P* < 0.05). No significant changes were detected after injection of the miR-137-3p NC, which had no significant effects on IR-induced CAPN-2 expression (*P* > 0.05).

### 3.3. Effects of the miR-137-3p/CAPN-2 Interaction on IR-Induced Hind-Limb Motor Dysfunction

To further clarify the regulatory roles of the miR-137-3p/CAPN-2 interaction *in vivo*, hind-limb motor function was assessed (Figure 2(f)). As expected, compared with baseline and sham-operated rats, all IR-injured rats showed obvious hind-limb motor dysfunction during the reperfusion period (*P* < 0.05). Compared with the time-matched injured rats in the IR group, the rats injected with the miR-137-3p mimic exhibited higher average Tarlov's scores, whereas those injected with the miR-137-3p inhibitor showed lower Tarlov's scores (*P* < 0.05). Likewise, in conjunction with the mRNA and protein levels of CAPN-2, rr-CALP2 injection reversed the improvement in motor function, indicated by the comparable Tarlov scores in the IR groups at all observed timepoints (*P* > 0.05). There were no detectable differences between the IR-injured rats treated with or without miR-137-3p NC at any of the observed time points (*P* > 0.05).

### 3.4. Modulation of CAPN-2 Expression and Activity by miR-137-3p in VSC4.1 Neurons after OGD/R

**Figure 3:**
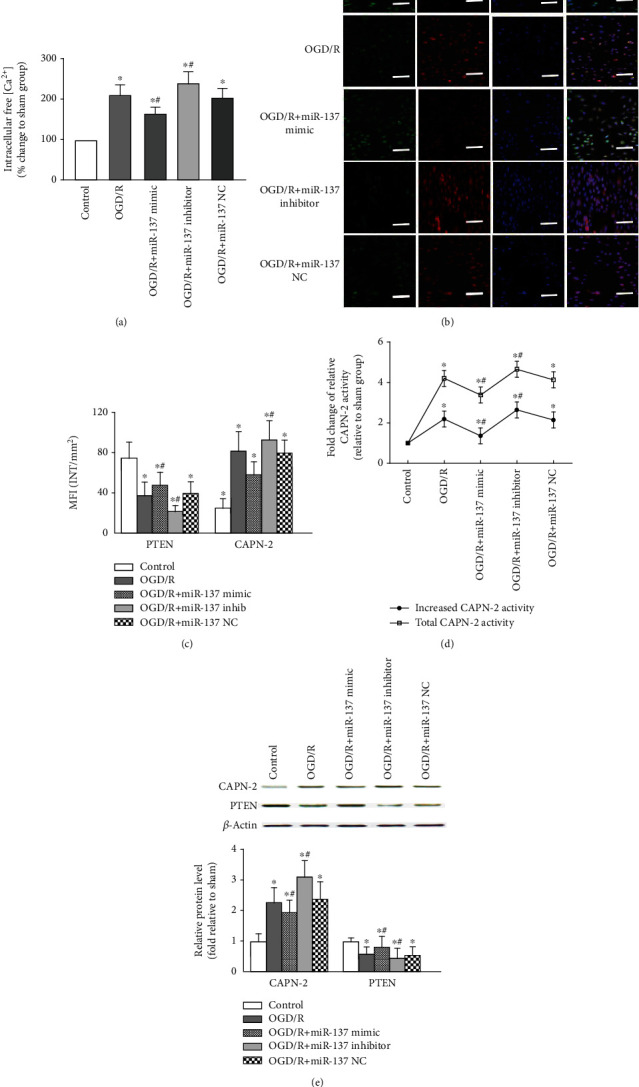
Modulation of CAPN-2 expression and activity by miR-137-3p in VSC4.1 neurons after OGD/R. (a) Percentage of intracellular free [Ca^2+^] in each condition after OGD/R. (b) Representative double immunofluorescence staining showing that PTEN (green) and CAPN-2 (red) are predominantly localized in the cytoplasms of VSC4.1 neurons. Scale bar = 50 *μ*m. (c) Quantification of the MFIs of PTEN and CAPN-2 in neurons of each treatment group. (d) Statistical analysis of total CAPN-2 and increased CAPN-2 activities at 24 h post OGD/R. (e) Representative Western blots and protein quantification of CAPN-2 and PTEN in neurons. All samples were analyzed in triplicate, and the data are expressed as the mean ± SD. ^∗^*P* < 0.05 versus the control group; ^#^*P* < 0.05 versus the OGD/R group.

Given that increased intracellular Ca^2+^ levels can activate CAPN-2 [13], we measured the free intracellular [Ca^2+^] in each treatment group at 24 h post OGD/R. As expected, compared to control cells, VSC4.1 neurons exposed to OGD/R for 24 h exhibited significantly increased intracellular free [Ca^2+^] (Figure 3(a), *P* < 0.05). In addition, miR-137 mimic pretreatment effectively prevented the OGD/R-induced [Ca^2+^] increase, whereas the miR-137 inhibitor aggravated the [Ca^2+^] increase (*P* < 0.05). No differences were detected between cells treated with or without miR-137 NC (*P* > 0.05).

Because PTEN is a selective substrate for CAPN-2 [32], the OGD/R-induced changes in CAPN-2 expression and activity were further confirmed by assessment of PTEN at the same observed time points. As shown by representative images of double fluorescent staining, both PTEN and p35 fluorescent labels were predominantly localized in the cytoplasms and nuclei of VSC4.1 neurons (Figure 3(b)). Consistent with previous studies [19, 32], the mean PTEN and CAPN-2 immunoreactivities exhibited opposite changes in all treatment groups, confirming that the net and total CAPN-2 activities were changed in accordance with the intracellular [Ca^2+^] (Figures 3(c) and 3(d), *P* < 0.05). Similar to the Western blot results *in vivo* and fluorescence quantification shown in Figure 3(c), the CAPN-2 protein levels were significantly decreased by miR-137 mimic treatment but increased by miR-137 inhibitor treatment (Figure 3(e), *P* < 0.05). Conversely, PTEN protein levels were increased in miR-137 mimic-transfected cells and decreased in miR-137 inhibitor-transfected cells (*P* < 0.05). No such changes were detected upon pretreatment with miR-137 NC (*P* > 0.05).

### 3.5. Modulation of p35 Cleavage and p25/Cdk5 Activation by the miR-137-3p/CAPN-2 Interaction after OGD/R

**Figure 4:**
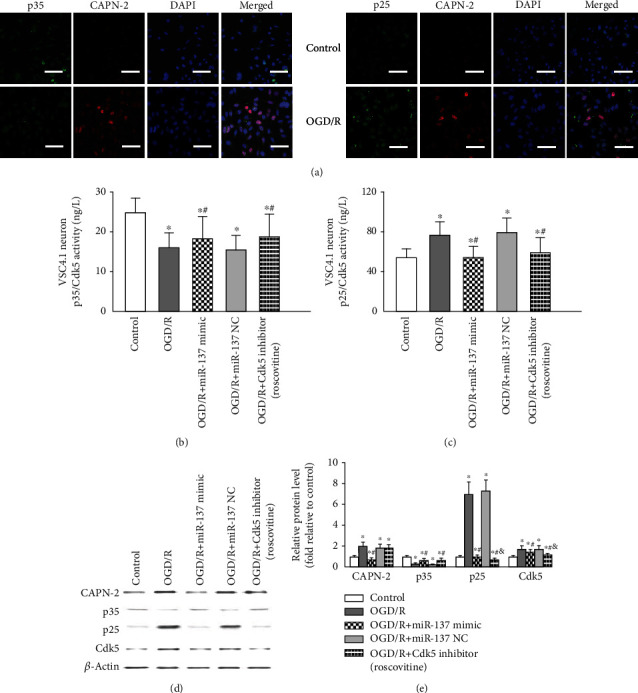
Modulation of p35 cleavage and p25/Cdk5 activation by the miR-137-3p/CAPN-2 interaction after OGD/R. (a) Representative double immunofluorescence staining showing that p35 (green) and CAPN-2 (red) (left panel) and p25 (green) and CAPN-2 (red) (right panel) are predominantly localized in the cytoplasms of VSC4.1 neurons. Scale bar = 50 *μ*m. (b, c) Quantification of p35/Cdk5 and p25/Cdk5 activities in VSC4.1 neurons of each treatment group by ELISA. (d) Representative Western blots and protein quantification of CAPN-2, p35, p25, and Cdk5 in neurons. All samples were analyzed in triplicate, and the data are expressed as the mean ± SD. ^∗^*P* < 0.05 versus the control group; ^#^*P* < 0.05 versus the OGD/R group; ^&^*P* < 0.05 versus the OGD/R+miR-137 mimic group.

Then, we tested whether p35 cleavage and subsequent p25/Cdk5 activation were regulated by the miR-137-3p/CAPN-2 interaction in VSC4.1 neurons by double immunofluorescence staining and Western blot, as previously published [5, 19]. As shown in Figure 4(a), representative fluorescence images showed CAPN-2, p35, and p25, and all were predominantly localized in the cytoplasms and nuclei of VSC4.1 neurons. OGD/R injury induced significant increases in CAPN-2 and p25 immunoreactivity but decreased p35 immunoreactivity in neurons at 24 h post injury, consistent with the Western blot results shown in Figures 4(d) and 4(e) (*P* < 0.05). Furthermore, the ELISA and Western blot results showed that, in contrast to the decrease in CAPN-2 expression caused by mimic transfection, transfection with the miR-137-3p mimic significantly reversed the OGD/R-induced decrease in p35 activity and protein expression (Figures 4(b)–4(e), *P* < 0.05), whereas no differences were detected in the presence of miR-137 NC (*P* > 0.05). As expected, following p35 cleavage, the activity and protein expression profiles of p25 and Cdk5 were changed in parallel to the CAPN-2 protein level detected in each treated group (*P* < 0.05).

Additionally, transfection with roscovitine, a specific Cdk5 inhibitor, regulated p35, p25, and Cdk5 protein levels and activity in a manner similar to that of miR-137-3p mimic transfection, just as roscovitine and the mimic had comparable effects on CAPN-2 expression (Figures 4(b)–4(e), *P* > 0.05).

### 3.6. Modulation of Caspase-8 Activation by the miR-137-3p/CAPN-2 Interaction after OGD/R

**Figure 5:**
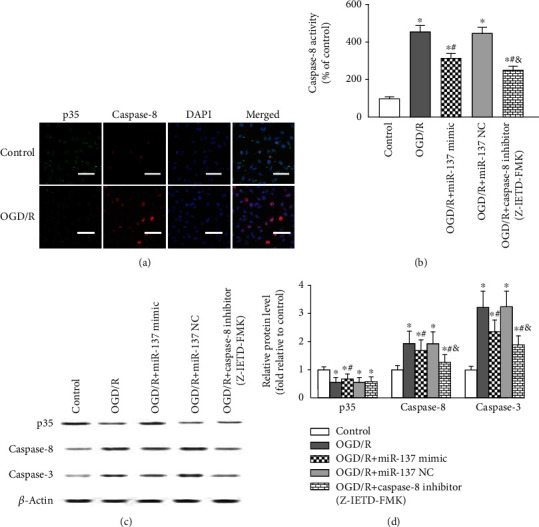
Modulation of caspase-8 activation by the miR-137-3p/CAPN-2 interaction after OGD/R. (a) Representative double immunofluorescence staining showing that p35 (green) and caspase-8 are predominantly localized in the cytoplasms of VSC4.1 neurons. Scale bar = 50 *μ*m. (b) Quantitative analysis of caspase-8 activity in VSC4.1 neurons of each treatment group. (c, d) Representative Western blots and protein quantification of p35, caspase-8, and caspase-3 in neurons. All samples were analyzed in triplicate, and the data are expressed as the mean ± SD. ^∗^*P* < 0.05 versus the control group; ^#^*P* < 0.05 versus the OGD/R group; ^&^*P* < 0.05 versus the OGD/R+miR-137 mimic group.

Likewise, we also assessed the regulatory effects of the miR-137-3p/CAPN-2 interaction on the caspase-8-mediated apoptotic network. As shown in representative fluorescent images, p35 and caspase-8 were identically localized in the cytoplasms of VSC4.1 neurons. OGD/R injury induced opposite changes in p35 and caspase-8, as it decreased the immunoreactivity and protein expression of p35 but increase the immunoreactivity and protein expression of caspase-8 at 24 h post injury (Figures 5(a), 5(c), and 5(d), *P* < 0.05). In contrast to the effects of mimic transfection on p35 expression, miR-137-3p mimic transfection significantly decreased the caspase-8 activity and protein levels (Figures 5(b)–5(d), *P* < 0.05), whereas miR-137 NC had no effect (*P* > 0.05). However, compared with that of the miR-137-3p mimic, transfection with Z-IETD-FMK, a specific caspase-8 inhibitor, had greater inhibitory effects on caspase-8 activity and protein levels at 24 h post transfection (Figures 5(b)–5(d), *P* < 0.05).

Additionally, the protein expression of caspase-3, the final executor of the apoptotic network, was measured in each treatment group at the same time point. The Western blot results showed that the change in caspase-3 protein expression was in accordance with that in caspase-8 protein expression (Figures 5(c) and 5(d)).

### 3.7. Modulation of VSC4.1 Neuronal Apoptosis by the miR-137-3p/CAPN-2 Interaction after OGD/R

**Figure 6:**
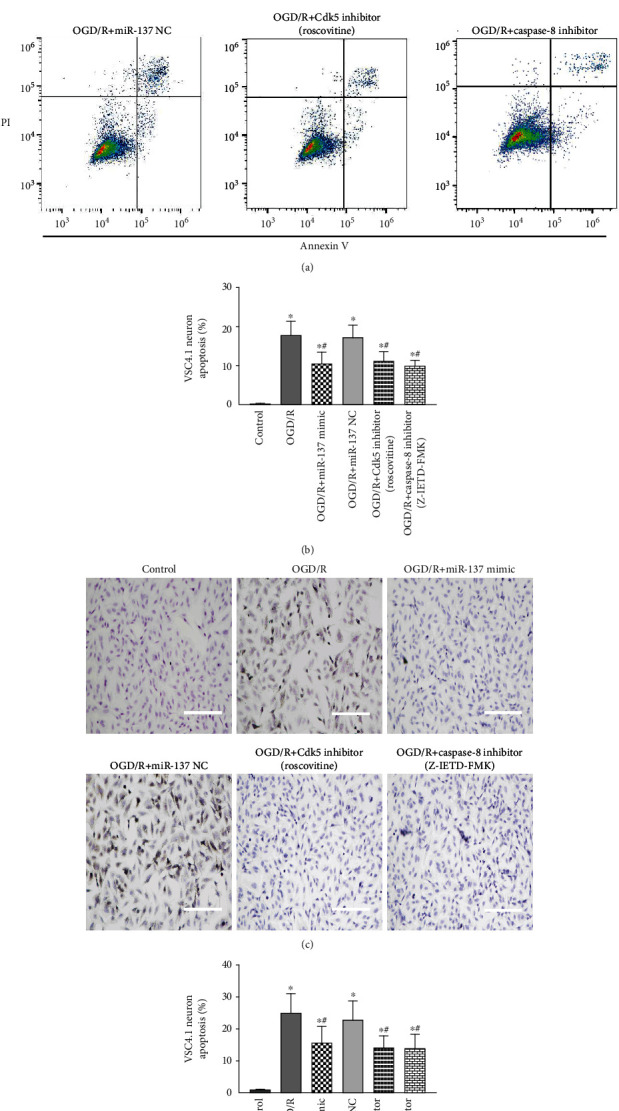
Modulation of VSC4.1 neuronal apoptosis by the miR-137-3p/CAPN-2 interaction after OGD/R. (a) The percentage of OGD/R-induced neuronal apoptosis in each treatment group as determined by flow cytometry. (b) Quantification of the percentage of apoptotic neurons. (c) Representative images of OGD/R-induced neuronal apoptosis in each treatment group as determined by the TUNEL assay. (d) Quantification of the percentage of apoptotic neurons. The cells detected with the brown color were regarded as positive, and the quantity was determined from six random fields. Scale bar = 50 *μ*m. All samples were analyzed in triplicate, and the data are expressed as the mean ± SD. ^∗^*P* < 0.05 versus the control group; ^#^*P* < 0.05 versus the OGD/R group.

Finally, the effects of the miR-137-3p/CAPN-2 interaction on neuronal apoptosis were assessed by flow cytometry *in vitro*. Consistent with the assessment of motor function *in vivo*, OGD/R insult obviously increased the percentage of apoptotic neurons (A2 + A4 quadrant) at 24 h after reperfusion (Figures 6(a) and 6(b), *P* < 0.05). Treatment with the miR-137-3p mimic, roscovitine, or Z-IETD-FMK had comparable and significant inhibitory effects on OGD/R-induced neuronal apoptosis (*P* < 0.05), whereas treatment with miR-137 NC had no such inhibitory effects (*P* > 0.05).

In addition, a TUNEL assay was performed to further validate and visualize the neuronal apoptosis. Consistent with the manufacturer's instructions, the representative apoptosis were neurons with tightly clustered brown staining (Figure 6(c)). And the quantification of the TUNEL-positive cells were changed in the same pattern as those in flow cytometry among the treatment groups, suggesting an important role of the miR-137-3p/CAPN-2 interaction in regulating subsequent Cdk5 and caspase-8 overactivation and neuronal apoptosis (Figure 6(d), *P* < 0.05). No significant differences were observed between the injured neurons treated with or without miR-137 NC (*P* > 0.05).

## 4. Discussion

Our previous studies revealed that IR-induced dysregulation of miR expression in spinal cords played important roles in driving pathogenesis during the reperfusion period and finally caused severe motor and sensory dysfunction [5, 6, 29]. Recently, an increasing number of studies have suggested that miR-based gene therapy is a promising treatment for neurological recovery by effectively preventing neuronal apoptosis. In the present study, we investigated the function and mechanisms of miR-137-3p and its target CAPN-2 in both *in vivo* and *in vitro* IR models to better understand the pathophysiological mechanisms and find better treatments in the clinic.

Previous studies have suggested prosurvival roles for CAPN-1 activation but destructive roles for CAPN-2 activation in retinal ganglion cell degeneration [17, 19]. However, none of the studies addressed the definite roles of calpain isoforms during the development of IR-induced pain hypersensitivity. In this context, we examined CAPN-1 and CAPN-2 protein expression and assessed motor function using the Tarlov scores at several time points post IR. Our results showed that only the temporal expression patterns of CAPN-2 were negatively correlated with IR-induced motor dysfunction, with initial significant differences being detected at 0.5 h post IR and peaking at 4 h post IR (Figure 1). This finding was consistent with that of a previous study on spinal cord injury, in which the progressively increased calpain content in the lesion was first detected as early as 30 min after trauma and increased by 91% at 4 h after trauma [36]. We further explored the cellular localization of CAPN-2 in major spinal cord cell types by double immunofluorescence at 4 h post IR when CAPN-2 expression reached its peak. Representative images and quantification showed that CAPN-2 was primarily expressed in spinal neurons, indicating that neuronal CAPN-2 might be the major effector during the reperfusion period.

MiRs are small RNA molecules that negatively regulate gene expression by binding with the 3′-UTRs of targets via complementary base pairs [28, 34]. MiRs are widely expressed in the CNS and have been implicated in multiple pathological processes, including IR [29]. We have suggested that some miRs, including miR-187-3p, miR-27, and miR-125b that are highly expressed in spinal cords, may provide new insights for research and clinical treatment [5, 6, 29]. Likewise, using miR microarray and luciferase assays, we herein found that miR-137-3p expression was substantially changed at 4 h post IR, and miR-137-3p exhibited a targeted interaction with CAPN-2 (Figure 2). Continuous intrathecal injection of synthetic miRs before IR was previously reported to effectively regulate miR expression and that of corresponding target genes *in vivo* [5, 6, 29]. Given the complicated cellular crosstalk *in vivo*, we defined the overall effects of the miR-137-3p/CAPN-2 interaction by assessing motor function in a rat model. As expected, in contrast to the decreased CAPN-2 protein expression, intrathecal injection of the miR-137-3p mimic substantially increased Tarlov's scores, indicating decreased motor dysfunction, whereas treatment with the miR-137-3p inhibitor or NC did not have these effects. Moreover, to further confirm the above interaction, exogenous CAPN-2 (rr-CALP2) was delivered directly via the intrathecal route. Consistent with the ability of exogenous CAPN-2 to activate intrinsic CAPN-2 in uninjured nerves [35, 37], the synergistic increase in CAPN-2 expression caused by exogenous rr-CALP2 injection was significantly prevented by the miR-137-3p mimic, as comparable CAPN-2 protein levels and similar behavioral assessments were observed throughout the reperfusion period in injured rats treated with or without combined injection with rr-CALP2 (Figure 2). These findings suggested that miR-137-3p acted as a functional regulator of CAPN-2 in the spinal cord.

As a trigger, CAPN-2 requires millimolar (0.250-0.750 mM) calcium concentrations for its activation [13]. Thus, parallel *in vitro* experiments were performed to better define the possible mechanisms by which the miR-137-3p/CAPN-2 interaction is implicated in VSC4.1 neuronal apoptosis. Consistent with the *in vivo* results and VSC4.1 neuron glutamate-related neurotoxicity [14], overactivation of CAPN-2 was accompanied by an increase in the intracellular free [Ca^2+^] in OGD/R-stressed VSC4.1 neurons; both the MFI of CAPN-2 and total and net CAPN-2 activities were significantly increased by OGD/R treatment (Figure 3). Previous reports have indicated that both peptide and nonpeptide CAPN inhibitors downregulate CAPN expression by preventing increases in intracellular free [Ca^2+^] [14, 38]. Consistently, our current data showed that the synthetic miR-137 mimic also prevented the increase in intracellular [Ca^2+^] and decreased CAPN-2 expression and activity in stressed neurons. In addition, PTEN is a selective substrate for CAPN-2 [32] and is thus widely used to quantitatively measure neuronal CAPN-2 activity *in vivo* and *in vitro* [19, 32]. As expected, PTEN and CAPN-2 were identically localized in the cytoplasms of neurons, but their protein levels and immunoreactivities changed in opposite directions in response to transfection with the different treatments. Decreased PTEN expression indicates an increase in CAPN-2 activation; therefore, these results suggest that OGD/R-induced CAPN-2 overactivation was regulated by synthetic miR.

Previous *in vitro* and *in vivo* studies have shown that CAPN-2 upregulation triggers neuronal apoptosis [20, 39]. These studies found that activated CAPN-2 directly and precisely cleaved its substrate, membrane-bound protein p35, into p25, which consequently resulted in Cdk5 activation in cultured primary neurons and retinal ganglion cells [13, 20, 39]. Similarly, our *in vitro* immunofluorescence staining (Figure 4) revealed that the cytoplasmic and nuclear labels for p35 and p25 fluorescence completely overlapped with the CAPN-2 labels in VSC4.1 neurons. In a previous study, during polybrominated diphenyl ether-153-induced neuronal apoptosis, p35 was found to accumulate in the perinuclear region and plasma membrane, and p25 was localized in both the cytoplasm and nucleus [20]. Given that the p35/Cdk5 complex mainly functions in the nucleus, these mislocalizations of p35 and p25 might signify the formation of the p25/Cdk5 complex [20, 40, 41]. Additionally, our Western blot results showed that the pattern of Cdk5 protein expression in each group changed in agreement with the protein levels of p25 and CAPN-2 and oppositely with the protein levels of p35 (Figure 4). On the other hand, the changes in p35 and p25 activities in being accordance with the protein level of CAPN-2 in neurons transfected with the miR-137-3p mimic or NC also demonstrated that the conversion of p35 into p25 requires CAPN-2. Additionally, selective inhibition of CAPN-2 expression has been shown to preserve both the structure and function of vulnerable neurons [14, 17, 19]. In this study, pretreatment with the miR-137-3p and Cdk5 inhibitors comparably inhibited the numbers of neurons located in the A4 and A2 quadrants of the flow cytometry dot-plot graphs and also the neurons with tightly clustered brown staining (referred as the TUNEL-positive cells) in the TUNEL assay after OGD/R insult (Figure 6). These findings all support the hypothesis that the dynamic localization of p35 and p25 is a marker of p25/Cdk5 activation-induced apoptosis.

Caspase cascade activation has been suggested to be central to neuronal apoptosis during IR injury [5, 9]. Acting as the apical member of the caspase family, caspase-8 overactivation has been shown to be especially important for controlling a series of broad caspase cascade networks by initiating the activation of downstream caspases, such as caspase-3 [24, 42]. Furthermore, as a cysteine protease, caspase-8 requires proteolytic cleavage before activation. Under normal conditions, caspase-8 forms the p35-caspase-8 complex via the formation of a covalent bond with the N terminus of p35 [26], which is known to be a major element necessary for preserving the p35-caspase-8 complex, and the baculovirus p35 protein has been demonstrated to effectively block the apoptosis cascade by preventing caspase-8 proteolysis and activation [24, 26]. In contrast, CAPN-2 overexpression-mediated p35 cleavage may cause conformational changes in p35 and consequently initiate aberrant caspase-8 activation. In agreement with the above hypothesis, a previous study showed that CAPN-2 was required to initiate endoplasmic reticulum stress-induced apoptosis mediated by a caspase-8-dependent pathway [25]. Similar to our *in vitro* immunofluorescence and Western blotting results, the fluorescent labels for p35 in the cytoplasm were identical to those for caspase-8 in neurons, and the protein expression levels of p35 and caspase-8 were changed in opposite directions when CAPN-2 expression was downregulated by pretreatment with the miR-137-3p mimic (Figure 5). In addition, our hypothesis that CAPN-2 initiates caspase-8-mediated apoptosis was additionally supported by similar results observed in neurons transfected with the miR-137-3p mimic and those transfected with the caspase-8-specific inhibitor Z-IETD-FMK. Both treatments exerted comparable inhibitory effects on the protein level and activity of caspase-8, the numbers of injured neurons in the A4 and A2 quadrants of the dot-plot graphs, and the amount of the TUNEL-positive cells (Figures 5 and 6). Acting as the final executor of the caspase family, caspase-3 expression was equally decreased in neurons transfected with the miR-137-3p mimic and Z-IETD-FMK, supporting the assumption that CAPN-2-induced caspase-8 activation may simultaneously lead to caspase-3 activation. Indeed, similar observations were made in a study of hydrogen peroxide-induced apoptotic pathways [25].

Of note, the CALP2-mediated mechanisms of pain hypersensitivity are somewhat divergent. First, CALP2 activity is not absolutely associated with reciprocal modulation of the CALP2 protein level. Interestingly, the addition of rr-CALP2 at 50 U/L induced significant decreases in the withdrawal thresholds of bilateral hind paws, but the differences in CALP2 expression increases on either side failed to reach significance at 3 d post drug administration [35]. Given the complicated *in vivo* crosstalk and signaling pathway network, CALP2-mediated cascade responses might be amplified by other factors in a positive feedback loop, such as the activation of nuclear factor-kappa B (NF-*κ*B) and the proinflammatory cytokine IL-6 [35]. Thus, it cannot be concluded from these data whether the increased CALP2 expression *in vivo* resulted from the activation of CALP2, NF-*κ*B-mediated inflammatory responses, or both or even by other signaling pathways, such as oxidative stress [43]. In addition, transfection with the Cdk5 inhibitor roscovitine had no significant effects on the expression of CAPN-2 or p35, possibly due to the eventual modulation of the CAPN-2/p35-p25/Cdk5 pathway [20]. Roscovitine is known to be highly specific for Cdk5 and is therefore unlikely to influence the activities of apical members of the signaling pathway [20]. Undoubtedly, whether caspase-8 directly activates caspase-3 by proteolytic cleavage or by the activation of caspase-3 activators needs to be elucidated in future studies.

## 5. Conclusions

This study highlights the roles of CAPN-2 in triggering motor dysfunction after spinal cord IR injury and investigates the target interactions with miR-137-3p both *in vivo* and *in vitro*. The effects of the miR-137-3p/CAPN-2 interaction on neuronal apoptosis may be attributed to CAPN-2 inhibition, which consequentially results in substrate p35 cleavage inhibition and thus prevents the overactivation of p25/Cdk5 and the initiation of the caspase-8-mediated caspase cascade.

## Data Availability

The materials supporting the conclusions of this article are included within the article.

## Abbreviations

BP: Blood pressure
BSA: Bovine serum albumin
Ca^2+^: Calcium ion
[Ca^2+^]: Ca^2+^ concentration
CAPNs: Calcium-activated neutral proteinases
Cdk5: Cyclin-dependent kinase-5
CNS: Central nervous system
EMEM: Eagle's minimum essential medium
FBS: Fetal bovine serum
HBSS: Hank's balanced salt solution
IF: Immunofluorescence
IR: Ischemia-reperfusion
miR: MicroRNA
MFI: Mean fluorescence intensity
MT: Mutant
NC: Negative control
pNA: P-Nitroaniline
PTEN: Tensin homolog
R: Ratio
SD: Standard deviation
VSC: Ventral spinal cord
UTR: Untranslated region
WT: Wild type.

## Supplementary Materials

Supplementary Materials Supplementary Figure: OGD/R-induced CAPN-2 upregulation and apoptosis in VSC4.1 neurons. (A) Representative double immunofluorescence staining shows that DAPI (blue), CAPN-2 (green), and Annexin V (red) are colocalized in the same neurons. Scale bar = 50 *μ*m. (B) Quantification of the number of CAPN-2-positive neurons with Annexin V. (C) Representative double immunofluorescence staining shows that DAPI (blue), CAPN-2 (green), and cleaved caspase-3 (red) are colocalized in the same neurons. Scale bar = 50 *μ*m. (D) Quantification of the number of CAPN-2-positive neurons with cleaved caspase-3. The data are expressed as the mean ± SD. ^∗^*P* < 0.05 versus the control group.
