# Supplementary material for: Involvement of the miR-137-3p/CAPN-2 Interaction in Ischemia-Reperfusion-Induced Neuronal Apoptosis through Modulation of p35 Cleavage and Subsequent Caspase-8 Overactivation
Source: Oxid Med Cell Longev. 2020 Dec 10;2020:2616871. doi: 10.1155/2020/2616871 (PMC7787780; doi:10.1155/2020/2616871)
Supplement: Supplementary Materials — Supplementary Figure: OGD/R-induced CAPN-2 upregulation and apoptosis in VSC4.1 neurons. (A) Representative double immunofluorescence staining shows that DAPI (blue), CAPN-2 (green), and Annexin V (red) are colocalized in the same neurons. Scale bar = 50 μm. (B) Quantification of the number of CAPN-2-positive neurons with Annexin V. (C) Representative double immunofluorescence staining shows that DAPI (blue), CAPN-2 (green), and cleaved caspase-3 (red) are colocalized in the same neurons. Scale bar = 50 μm. (D) Quantification of the number of CAPN-2-positive neurons with cleaved caspase-3. The data are expressed as the mean ± SD. ∗P < 0.05 versus the control group. [file 2616871.f1.pdf]

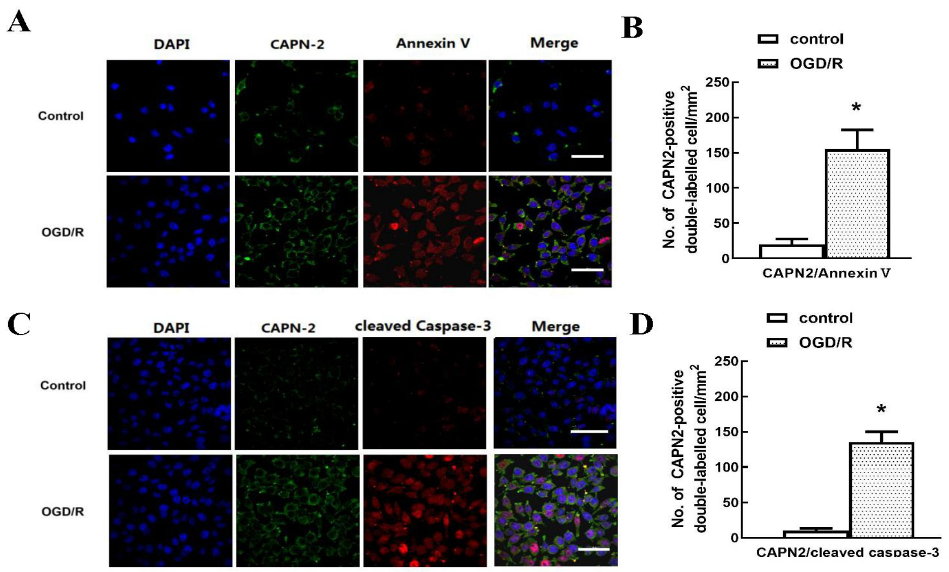

Supplementary Figure. OGD/R-induced CAPN-2 upregulation and apoptosis in VSC4.1 neurons. A, Representative double immunofluorescence staining showed that DAPI (blue), CAPN-2 (green) and Annexin V (red) were colocalized in the same neurons. Scale bar = 50 μm. B, Quantification of the number of CAPN-2-positive neurons with Annexin V. C, Representative double immunofluorescence staining showed that DAPI (blue), CAPN-2 (green) and cleaved caspase-3 (red) were colocalized in the same neurons. Scale bar = 50 μm. D, Quantification of the number of CAPN-2-positive neurons with cleaved caspase-3. The data are expressed as the mean±SD. *$P < 0.05$ versus the control group
